# Edible additive effects on zebrafish cardiovascular functionality with hydrodynamic assessment

**DOI:** 10.1038/s41598-020-73455-9

**Published:** 2020-10-01

**Authors:** Yu-Fang Wang, I.-Wei Chen, Satishkumar Subendran, Chun-Wei Kang, Bivas Panigrahi, Tzu-Fun Fu, Chia-Yuan Chen

**Affiliations:** 1 Department of Medical Laboratory Science and Biotechnology, National Cheng Kung University, No. 1 University Road, Tainan, 701 Taiwan; 2 Department of Mechanical Engineering, National Cheng Kung University, No. 1 University Road, Tainan, 701 Taiwan

**Keywords:** Biotechnology, Engineering

## Abstract

Food coloring is often used as a coloring agent in foods, medicines and cosmetics, and it was reported to have certain carcinogenic and mutagenic effects in living organisms. Investigation of physiological parameters using zebrafish is a promising methodology to understand disease biology and drug toxicity for various drug discovery on humans. Zebrafish (*Danio rerio*) is a well-acknowledged model organism with combining assets such as body transparency, small size, low cost of cultivation, and high genetic homology with humans and is used as a specimen tool for the in-vivo throughput screening approach. In addition, recent advances in microfluidics show a promising alternative for zebrafish manipulation in terms of drug administration and extensive imaging capability. This pilot work highlighted the design and development of a microfluidic detection platform for zebrafish larvae through investigating the effects of food coloring on cardiovascular functionality and pectoral fin swing ability. The zebrafish embryos were exposed to the Cochineal Red and Brilliant Blue FCF pigment solution in a concentration of (0.02‰, 0.2‰) cultured in the laboratory from the embryo stage to hatching and development until 9 days post fertilization (d.p.f.). In addition, zebrafish swimming behaviors in terms of pectoral fin beating towards the toxicity screening were further studied by visualizing the induced flow field. It was evidenced that Cochineal Red pigment at a concentration of 0.2‰ not only significantly affected the zebrafish pectoral fin swing behavior, but also significantly increased the heart rate of juvenile fish. The higher concentration of Brilliant Blue FCF pigment (0.2%) increased heart rate during early embryonic stages of zebrafish. However, zebrafish exposed to food coloring did not show any significant changes in cardiac output. The applications of this proposed platform can be further extended towards observing the neurobiological/hydrodynamic behaviors of zebrafish larvae for practical applications in drug tests.

## Introduction

A food additive (FA) is an add-on substance that can be nourishment or chemical preservative which turns out to be part of a food product either legitimately or by implication during some periods of processing, storage, and handling^1^. FAs are purposely added to enhance appearance, flavor, or for preserving the food^2^. Unfortunately, excessive intake of these additives has a detrimental effect on the human body. Thus, the toxicity of these additives used in food has raised critical attention among the consumers. It has been previously reported that patients with cerebral palsy experienced adverse effect due to intake of blue food additives, which can later develop the symptoms causing cyanosis^3^. The European Parliament and the Council published the guideline on FAs setting up that the lethal quality of FAs by the European Food Safety Authority, which intends to explicitly examine the toxicity of food coloring and provide information on food evidence that additives, may adversely affect the human body^2^. Owing to the hazardous effects, these food additives are prohibited in the United States, Japan, and some European nations^4,5^. In addition, the potential risks of food colorings are inconclusive. Past research has indicated that some food coloring can cause neurotoxicity in mice, genetic toxicity, and effects on the reproductive system^6^.

Considerably, there have been efforts to structure conventions that integrate zebrafish testing with different ways to deal with and assess the potential for synthesized substances to interface the cardiac and the behavioral systems. As zebrafish is an invaluable animal that has been broadly used as a genomic model in light of its hereditary comparability 70% to humans^7–9^, it is widely used to evaluate the toxicity of chemicals^10,11^ as well as biohazardous materials. Zebrafish being a vertebrate is close-by the mammalian emergence, undeniably appropriate for laboratory experimentation due to its expeditious development, optical transparency, and the easy phenotypic assessment through the chorion. Embryonic developmental process is accomplished within 72 h post fertilization (h.p.f.) and the cardiovascular system, liver, gut and kidney are well-developed by 96 h.p.f.^12^. According to previous studies, there is a developmental analogy between the embryonic zebrafish heart and the human heart^13^. Moreover, in vivo toxicology evaluation with zebrafish can be accomplished within a week, which a lot shorter period than that necessary when performing practically identical mammalian examinations^14^. As opposed to rodents, the zebrafish larvae are closer to the adolescent state in that the nervous system is mature and the fundamental organs are completely evolved during the examination. Furthermore, considerably very less amount of additives is needed for screening, as the larvae can be alive in less than 50 µL of fluid^15^. Although the use of the zebrafish as an ideal animal model paves the way to biological advances, the current progress of zebrafish tests is hindered with difficulty especially during the imaging. As of now, most researchers need to perform the positioning control operations on larvae to facilitate micro-scale screening and a broader view of imaging for zebrafish through manual means which may lead to unavoidable fish damage and eventually it can turn out with low efficiency and time-consuming for the entire imaging process^16^. In this aspect, an automated live imaging platform is in the current demand which will reduce the chance of inducing permanent damage to the zebrafish larvae during toxicity screening.

The microfluidic device breaks through the limitations of conventional methods such as significantly reducing the damage to fish and no need for anesthetics while observing the zebrafish. This significantly reduces the cost of the inspection, and easy for drug administration to inspect the development of organs in zebrafish with a higher survival rate^17–19^. In this context microfluidic technologies enact a promising direction that can support the development of the next-generation systems for phenotype-based physiological analysis^20^. Incited by rapidly increasing applications of zebrafish models in biomedicine and drug discovery, the microfluidic systems for these analysis have experienced remarkable attention in recent years^21^. The corresponding microfluidics were designed to meet the needs of practical applications. This validates that a microfluidic observation platform can be a good drug inspection system^22^. Using the microfluidic device as a drug-testing platform can not only perform physiological tests on zebrafish but also observe its behavioral capabilities during the drug testing.

The present study initialized a novel microfluidic paradigm built on a framework that was aimed to quantify and evaluate the toxicological effects of the food additives for in-vivo assessing on zebrafish model specimen. The zebrafish heart, with its similarity to the human heart, is a unique system for modeling both genetic and drug-driven cardiovascular investigations. This study discussed the investigation of zebrafish cardiovascular functionality on exposure to the commonly used food additives Cochineal Red and Brilliant Blue FCF. The study revealed that both food additives induced change in the cardiac response in a dose-dependent manner. In addition, spatiotemporal behavioral effects induced due to the instantaneous change in heartbeat caused by the food additives of zebrafish was visualized and quantified in a systematic manner as delineated in the following sections.

## Methods

### Microfluidic device fabrication

**Figure 1:**
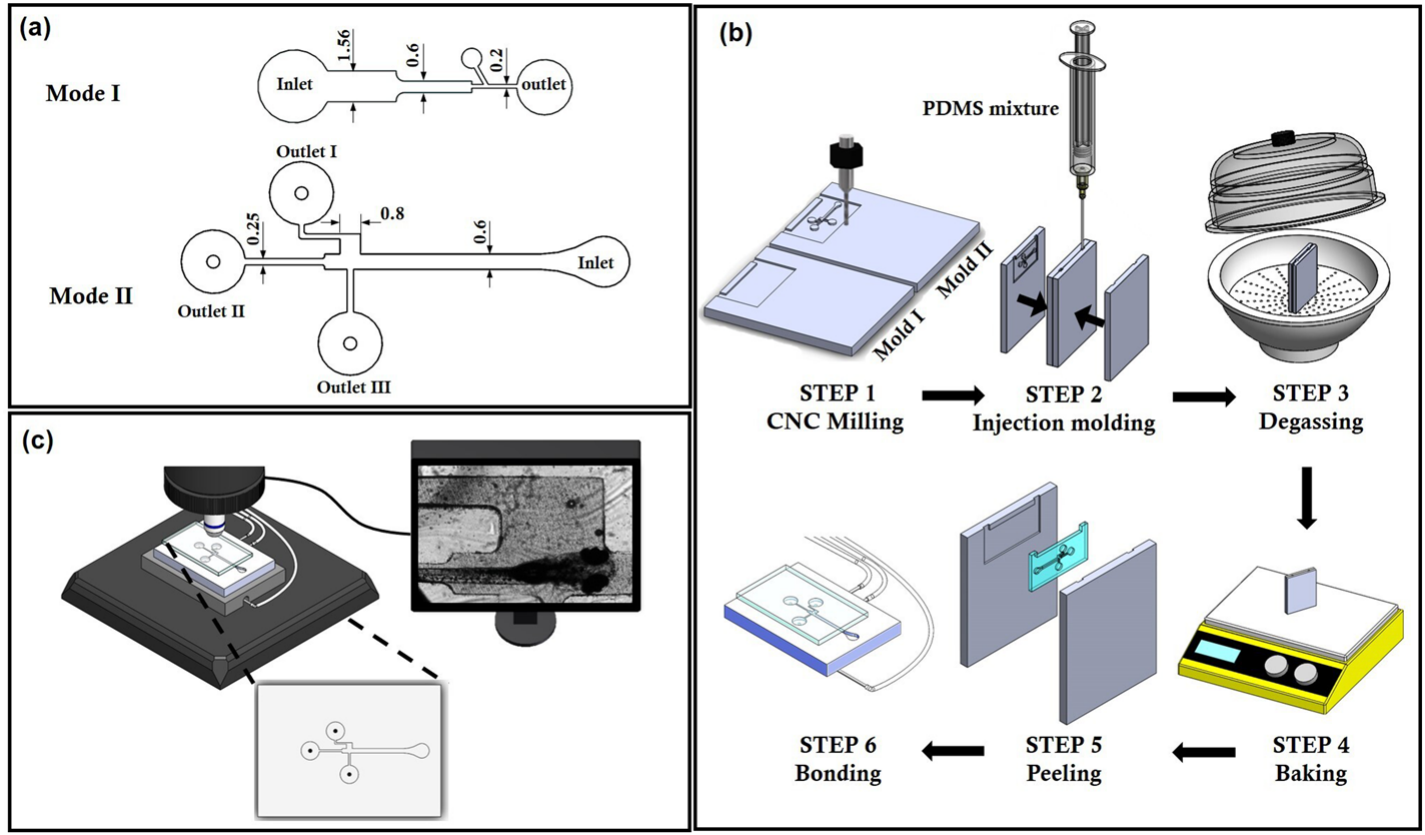
Design, fabrication, and the experimental test set-up of the microfluidic device to assist the hydrodynamic assessment of cardiovascular and the behavioral functionalities in zebrafish. (**a**) The description of the geometric design of the microfluidic device for observing cardiac functionality and pectoral fin swing behaviors of zebrafish. (**b**) Schematic illustration of the series of techniques involved in the microfluidics fabrication process. (**c**) An experimental observation framework for heart and behavior imaging of zebrafish in the microfluidic device without anesthetic treatment. Dimensions are in mm.

The design details of the microfluidic device used to quantify the heart rate and the pectoral fin beating are depicted in the Fig. 1a,b. The fabrication process of the microfluidic device consists of a series of computerized numerical control micromachining processes with the polydimethylsiloxane (PDMS) casting process. Firstly, the geometric design of the microfluidic device was inscribed on an acrylic substrate using a micro-milling technique. The molds were affixed to fill up with the mixture of PDMS and curing agents weighing in a ratio of 10:1. Further, the degassing process was carried out by following the curing process through hot plate baking at 95 °C for 48 h. Finally, the device was peeled from the parent mold^23–25^, as shown in Fig. 1.

### Zebrafish culture

Transgenic zebrafish larvae Tg (Cmlc2: eGFP/H2A: mCherry) were used and their fertilized eggs were raised and cultured in edible additive solutions at the temperature ranging 28 °C ± 1 °C in a light: dark cycle of 14 h:10 h cycle to maintain a natural circadian rhythm. All experiments were performed under the relevant laws and institutional guidelines set by the National Cheng Kung University Institutional Animal Care and Use Committee (IACUC) with approval number: 106313.

### Food pigmentation preparation

In this experimentation, two food additives commonly used in foods were selected, namely, Cochineal Red and Brilliant Blue FCF. They were diluted from the 2% weight concentration of the original solutions. The two-weight concentrations, 0.02‰, and 0.2‰ were brought into the zebrafish treatment in the microchannel. The zebrafish embryos of 4 h.p.f. were exposed to the daily refreshed embryo water containing food color additives for 6 days before further analysis and data collection. Besides, the control group was treated with fish water in E3 medium respectively.

### Zebrafish cardiovascular imaging and processing

To obtain a clear image of the zebrafish heartbeat for quantification, the zebrafish body was immobilized through the microchannel to avoid the movement of the zebrafish during the imaging. Under a stereomicroscope (Leica Z16 APO, Leica Camera AG, Wetzlar, Germany) equipped with a digital camera (Canon EOS 650D, Canon Inc., Tokyo, Japan), heartbeat images were recorded at 30 fps and then stored in a computer for further processing. The heartbeat images of the zebrafish were captured at 20× magnification for 20 s and recorded under a light source to observe the heart wall displacement for the estimation of the heart beats. The original color image was first converted into a negative mode to highlight the fluorescent heart of the tested zebrafish. Grayscale and brightness of the images were adjusted and converted into binary images, and clear and smoother heart wall boundary was obtained from the images for heartbeat measurement. Sampling points were selected from the heart wall boundary with movement recorded in the time domain. Further the collected time-dependent data were processed in the frequency domain to identify the dominant frequency serving as the heartbeat information in the quantitative manner. Specifically, the displacement of the heart wall of the zebrafish was the time-series signal. This determined time series signal was transformed into a frequency spectrum using the Fast Fourier Transform (FFT) algorithm through the in-house MATLAB programming for the calculation of the power spectral density (PSD). The dominant frequency in PSD corresponded to the heart rate of zebrafish.

### Zebrafish swimming behavior and high-speed imaging of the pectoral fin beats

The swimming behavior, including distance and acceleration were collected using DanioVision (Noldus, Wageningen, The Netherlands), a high-throughput image recording and analysis system. Larvae of 4 h.p.f. were exposed to the food color additives for 6 days and placed in a 48 well-plate with one larva per well. The plate was incubated in the detecting chamber for 10 min before data collection. Larval swimming path was video-tracked and recorded for 30 min at the speed of 10 fps. Data were analyzed using the build-in software EthoVision XT.

To provide more scientific evidences on how the food additives may affect the cardiovascular functions of zebrafish specifically in terms of swimming behaviors, a flow visualization technique was employed, namely, micro-particle image velocimetry (µPIV). First, to reduce the harmful effect that may be caused by a necessary component for fluid tracking, the fluorescent particles were replaced by smashed egg yolk particles with a better biocompatible property. The cooked egg yolk weighing 0.04 g was mashed thoroughly in 1.5 mL of DI water inside a conical tube. Further, vortexed the diluted egg yolk mixture for about 1–2 min until it appears as a homogenous mixture. The prepared egg yolk particle solution was infused into the designed microfluidic device for the observation of the induced fluid flow pattern disturbed by zebrafish fin beating. Images were obtained using an optical microscope (BX60, Olympus Corp., Japan) and a high-speed camera (NX4-S2, IDT, Tallahassee, FL, USA). The quantified flow fields were calculated by a PIV software package (Dynamic Studio, Dantec Dynamic A/S, Denmark), and the results are presented as the calculated velocity vectors overlapped with the vorticity contour map. The first-pass PIV interrogation window was 64 × 64 pixel with 50% overlap for 10 iterations, and the second-pass was 32 × 32 pixel interrogation size with 50% overlap for 10 iterations. Grid interpolation was used to increase the vector distribution and effectively reduce the bad vector and outliers.

### Statistical methods

To compare the effects of different food additives on the cardiovascular responses of zebrafish, data analysis was performed using a statistical software (SPSS Statistics, IBM, United States). Two-way ANOVA was used to analyze experimental data and was performed multiple comparison tests using the Scheff method. The factors affecting the final heartbeat results include the food additive types and concentration together with the number of days after the fertilization of zebrafish eggs. The control group refers to zebrafish without food additive treatment. In the comparison of the induced flows by pectoral fin beats, a two-tailed independent sample t-test was selected to compare the significant difference among the control group and the food colorant groups.

## Results

**Figure 2:**
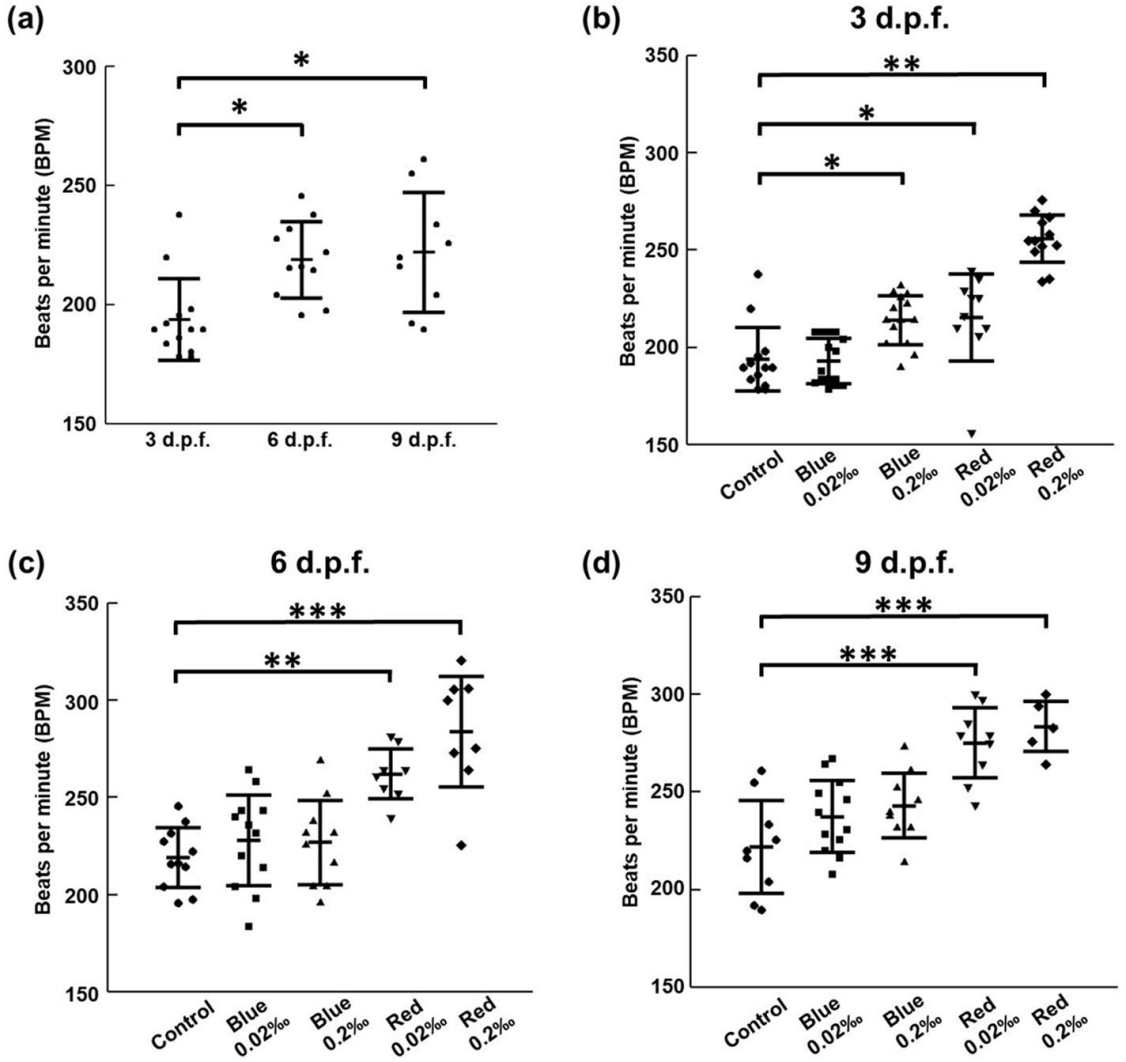
Assessment of the cardiac rhythm of zebrafish larvae treated with food additives. (**a**) The heartbeat rates of zebrafish larvae (3, 6, and 9 d.p.f.) determined before the exposure to the food additive is the control group (*p < 0.05). (**b**–**d**) Heartbeats were recorded from a zebrafish larvae (3, 6, and 9 d.p.f., respectively) treated with varying concentrations of food additives and their comparison with the control group. Cochineal Red additives with 0.2‰ concentration caused an increase in the heartbeat during the early developmental stages of zebrafish larvae. Heart beats per minute are expressed as means ± SD (*p < 0.05, **p < 0.01 and ***p < 0.001).

In the initial tests, lower concentrations were used for the assessment of the cardiovascular functionality of the food additives in the transgenic zebrafish strain with Cochineal Red (0.02‰) and Brilliant Blue FCF (0.02‰). Zebrafish treated with food additives showed a first significant increase in heart rate of 232.2 ± 23.68 bpm (beats per minute) at 3 d.p.f. for Cochineal Red (0.02 ‰) whereas the decrease heart rate of about 192.72 ± 12.01 bpm was observed for Brilliant Blue FCF (0.02‰) as shown in Fig. 2. Figure 2a represents the heartbeat rates of zebrafish larvae (3, 6, and 9 d.p.f.) without the exposure to the food additive used as the control group to further compare the heartbeat rate with those exposed to varying concentrations of food additives. It was observed that the heartbeat rate for zebrafish larvae (3, 6, and 9 d.p.f.) where 193 ± 17.09, 218.78 ± 16.02, and 221.80 ± 25.17, respectively. Additionally, in later tests, higher concentration treatments of Cochineal Red were also utilized to examine the heart rate variations at different developmental stages of zebrafish. Notably, zebrafish treated with Cochineal Red (0.2‰) showed a significant stimulating effect in heart rate of about 255.65 ± 12.72 bpm, 283.73 ± 30.54 bpm, 283.32 ± 14.31 bpm for 3, 6 and 9 d.p.f., respectively, which resulted in a higher slope from 3 d.p.f. to 6 d.p.f. in early stages as represented in Fig. 2b–d, respectively. Consecutively, for higher concentration treatments of Brilliant Blue FCF (0.2‰) consistently induced an increase in heart rate from 213.81 ± 12.93 bpm, 226.62 ± 22.76 bpm to 242.8 ± 17.6 bpm for 3, 6, and 9 d.p.f.. A more stable increase over time was found in the Brilliant Blue FCF (0.2‰) test. The overall effect of continuous food additive treatment in general increased the heart rate.

It was prominently noted that the heart rate was increased in a dose-dependent manner in zebrafish larvae (3 d.p.f.) exposed to Cochineal red additives for both concentrations compared to the control group (232.2 ± 23.68 bpm, **p < 0.01 and 255.65 ± 12.72 bpm, **p < 0.01). The average heartbeat rate for 6 d.p.f. trials exposed to a higher concentration of Cochineal Red additives on comparing with the control group set out (283.73 ± 30.54 bpm, ***p < 0.001). Cochineal Red additives elicited a significant boost in heartbeat rate as compared to that of the control group. In younger zebrafish larvae (3 d.p.f.), the effect of the Brilliant Blue FCF pigment application resulted in no major change of heart rate but it was verified that the expeditious increase in the concentration of food additives does affect the heartbeat of zebrafish in later developmental stages. Thus, larvae exhibited variant results in the control group at all concentrations studied.

To assess cardiovascular functionality during the early developmental stages, zebrafish larvae (3 d.p.f.) exhibited a subtle response to both the food additive treatments. Besides, during the mature stages of zebrafish larvae (9 d.p.f.) stability still high heart rate value was achieved in response to the food additives. Over time, intervals food pigment treated zebrafish larvae (9 d.p.f.) showed insignificant change in the progression of heart rate. It was also observed that zebrafish exposed to food pigmentation differ in appearance. In addition, significant occurrence of abnormal circulation, cardiac malformation, arrhythmia, or pericardial edema observed in all selected developmental stages were not clearly observed.

**Figure 3:**
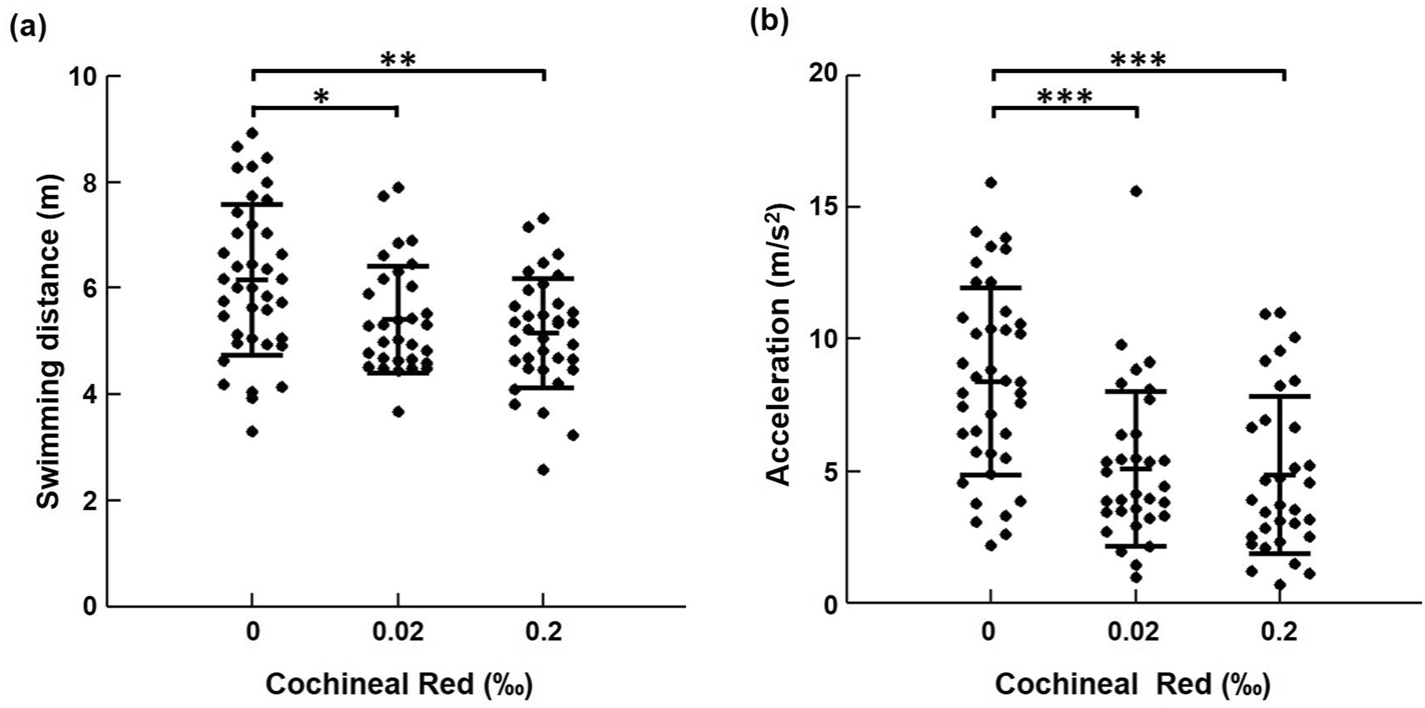
The effects of food additive concentration on the behavioral changes of zebrafish. (**a**) Effects of cochineal red pigmentation on swimming activity and (**b**) effects of cochineal red pigmentation on the acceleration of the zebrafish. Values that are significantly different from the control are indicated by asterisks (*p < 0.05, **p < 0.01 and ***p < 0.001).

Considering swimming speed, Larval swimming path was video-tracked and recorded for 30 min at the speed of 10 fps. Statistically, significant effects were observed for Cochineal Red additives compared to the control as depicted in Fig. 3. The average swimming distance of 514.92 cm was recorded for zebrafish larvae (6 d.p.f.) exposed to the higher Cochineal Red concentration (0.2‰) tended to swim slower than those from water control which set out to be 615.15 cm with significant differences (**p < 0.01). Average swimming distance for lower concentrations (0.02‰) was set out to be 539.96 cm which is higher than the distance recorded for the higher concentration of the Cochineal Red additives, significant differences were found (*p < 0.05) on comparison with the control. Unlike the Cochineal Red (0.2‰) treated fish, Cochineal Red (0.02‰) treatment produced higher acceleration speed of 507.19 cm/s^2^. Statistically significant differences concerning swimming distance and swimming acceleration were found between water-controlled zebrafish and food additive exposed zebrafish. It was observed that zebrafish exposed to Cochineal Red additives for a long time not only affected their motility but also increased concentration led to a decline in the mobility of zebrafish larvae.

**Figure 4:**
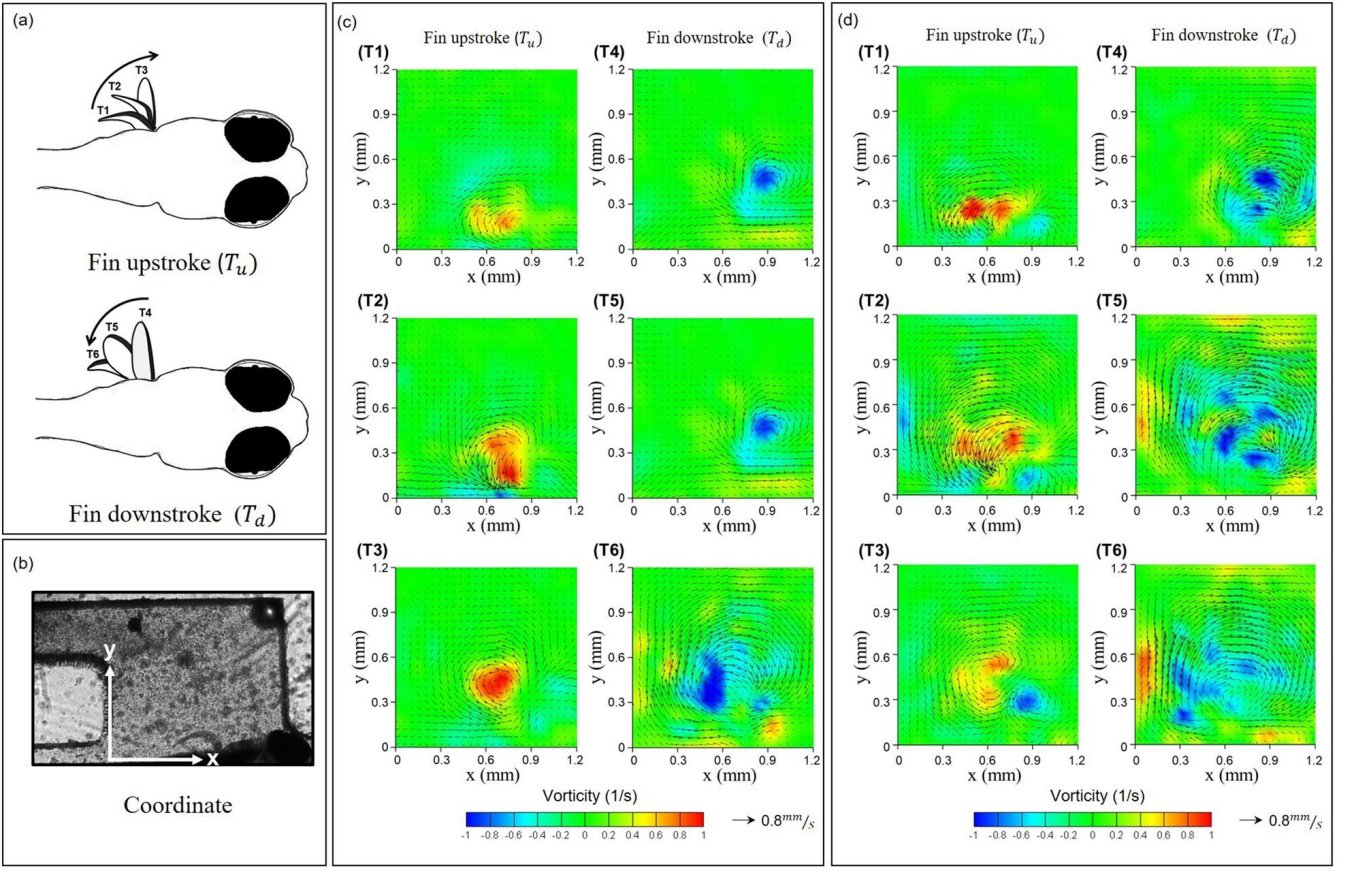
Hydrodynamic quantificational measures of zebrafish pectoral fin movements (**a**) pictorial representation of zebrafish pectoral fin movements (**b**) experimental image of zebrafish pectoral fin movements (**c**) for the control group and (**d**) the Cochineal Red pigment 0.2‰ group. The vorticity contour is super-imposed with the velocity vector field (black arrows) generated during pectoral fin beatings (**a**) upstroke and (**b**) down-stroke of zebrafish larvae (6 d.p.f.).

Further, a practical hydrodynamic test scheme was employed to identify the effect of the red food additive on the behavioral changes in zebrafish. First, the right pectoral fin and tail of the zebrafish were both immobilized inside the microchannel as shown in Fig. 4. Without the additive treatment, a forward vortex flow was generated around the pectoral fin region in the power stroke period. When the pectoral fin recovered, a vortex was generated above the pectoral fins in the revised manner. The muscle system of the fish can generate forces, which cause body deformations. The resulting motion produced a flow field in the surrounding water.

**Figure 5:**
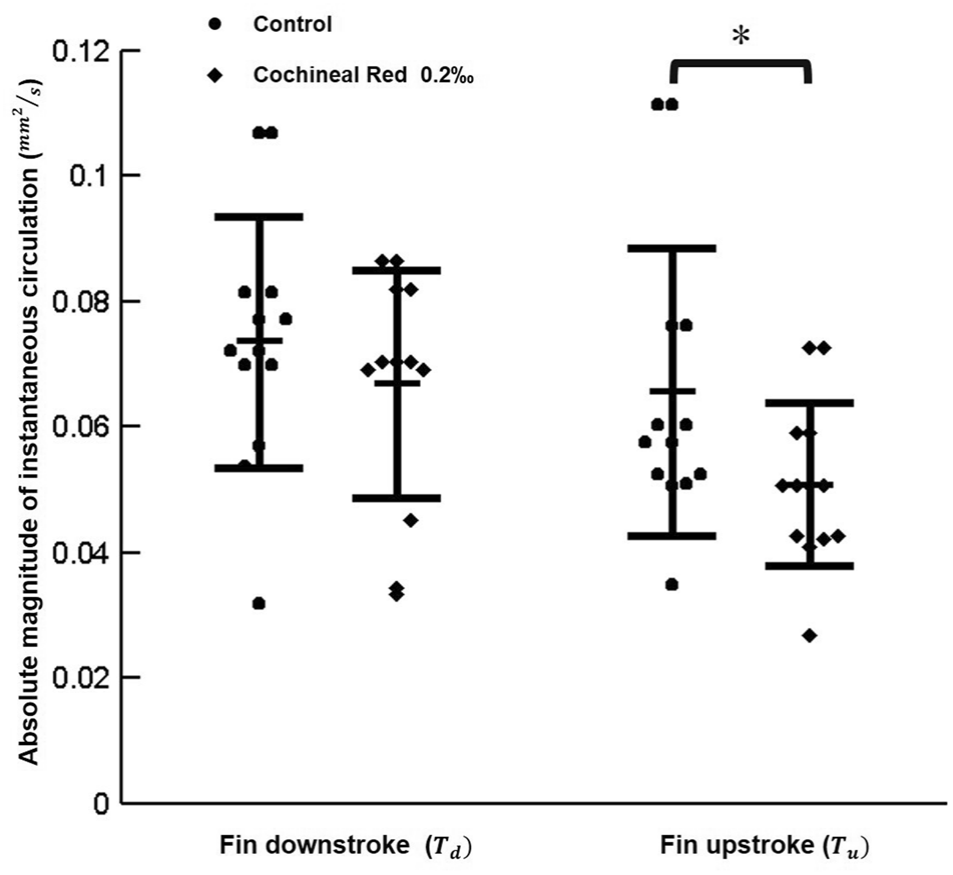
The absolute magnitude and instantaneous circulation formation due to the pectoral fin beating of zebrafish larvae (*p < 0.05) hydrodynamically with effective test samples in each experimental group (N = 15).

It was noted that zebrafish (6 d.p.f.) fins exposed to Cochineal Red additives relatively produced smaller magnitude of instantaneous circulation flux as compared to the untreated zebrafish in the control group (*p < 0.05) as depicted in Fig. 5. The adverse effect of the pigment exposure which may affect the pectoral fin motions. Throughout the test scheme it is possible to provide the quantitative results depicting the actual effects of red additives on zebrafish. This finding is important as it provided another evidence on identifying the pathway of the role of pigment chemicals in the cardiovascular system of zebrafish, which is beneficial for following up discussion on the safety level of these additives in human body.

## Discussion

The primary goal of this study was to assess whether food additives have similar influences with other additives in human through measuring cardiovascular functions of zebrafish larvae. Additives or drugs may induce cardiac repolarization by inhibiting heart rate effects in patients with concurrent cardiac risk factors can emerge into life-threatening extremity. This is currently a major source of attrition in drug development that has been a dominant reason to follow up specific ICH guidelines^26^. Thus, it can be very beneficial to perform the in vivo screening of drugs/additives for their toxicity using the zebrafish model. As a proof of concept, in this study the in-depth investigation was performed to reveal the early effects induced by the intake of food additives in zebrafish. To begin with, this study validated the applicability of the designed microfluidic device for real-time monitoring of heart rate variations in zebrafish larvae.

Previous studies have confirmed that tricaine and Diacetyl monoxime reduced the cardiac rate of zebrafish larvae^27^. In contrast, exposure to food pigment treatments increased the cardiac rate in zebrafish. As it was observed that exposure to Cochineal Red additives significantly affected the heart rate of zebrafish, which also led to changes in spontaneous movements and locomotor variations. Much of the work examining the behavior of fish at early developmental stages has been on the larval zebrafish which is been used to investigate the motor control and movements^28,29^. Zebrafish beats its pectoral fins, alternating itself rhythmically in combination with body undulation during slow swimming whereas during faster swimming, and the body undulates but the pectoral fins remains positioned close to the body^30,31^. Studies on pectoral fins have demonstrated that zebrafish increases its swimming speed by increasing fin beat frequency and amplitude^32^. Most certainly, further studies are essential.

The formation of the flow field in the circumferential area of the pectoral fin led again to deform the fluid forces, by creating a loop of interactions between the water and the body of the fish^33,34^. At the beginning of the power stroke, the fin started to accelerate backward and rotated clockwise with a positive angular acceleration. A thrust is produced by the created pressure difference induced by the flow disturbance. On the other hand, new layers of positive vorticity around the leading edge of the fin are formed. Early in the observation period, the higher concentration of Cochineal Red produced fewer circling behavior, suggesting that this higher concentration was effective in reducing the circling behavior. Measurements of circling, swimming activity, and preference all indicated food additive exposure associated the modification of these behaviors in zebrafish. Because of the decrease in circling behavior observed, it is hypothesized that Cochineal Red additives would also decrease overall swimming activity and that there would be a dose–response relationship. An additionally comprehensive study in this regard is still necessary. Zebrafish larvae during early developmental stages reach relatively high swimming speeds by beating their fins and tails with high frequencies and large amplitude. To generate these fast motions, the muscle system needs to produce high strain rates, and activate and deactivate at a high frequency^31^. But as the zebrafish larvae grow, fin and tail beat frequencies are decreased owing to the increasing body inertia. In addition, the presented analytical paradigm in evaluating the effects of drugs and chemicals on living animal models through microfluidics can be employed as a protocol for other tests of interest when a comprehensive cardiovascular and behavior functions need to be examined in a systematic manner.

## Conclusion

The zebrafish is a favorable alternative for biosafety investigational studies due to its small in size, hereditary characteristics, higher breeding capabilities, and most importantly, due to the similarities and physiology with that of humans. Thus, zebrafish as a model for assessing cardio-neuro destructiveness of assistance is an intellect of its focal points over other animal models. In this work, the adverse effects of food additives on the cardiovascular functionality of the zebrafish were enlighten. The average heartbeats with the 0.2‰ concentration of the Brilliant Blue FCF additives were 10% higher than that of the control group. Consecutively, the average heartbeats with the 0.2‰ concentration of the Cochineal Red additives were 32% higher than that of the control group. Regardless of food additives, the average heartbeats in the higher concentration group were 10% higher than those in the lower concentration group. The discussion of these findings can be further extended to the comprehensive hydrodynamic analysis of pectoral fins of zebrafish. In spite of the fact that the additional tests are in the pipeline, it is as of now obscure how food additives affect zebrafish. Still, the deliberate outcomes displayed in this examination provide a new look in terms of using the microfluidics for a combined biological and mechanical test for biosamples. The procured information acquired from this investigation will surely improve the current drug testing and toxicity practices for new medication disclosure and advancement.
